# Elastic transformation of histological slices allows precise co-registration with microCT data sets for a refined virtual histology approach

**DOI:** 10.1038/s41598-021-89841-w

**Published:** 2021-05-25

**Authors:** Jonas Albers, Angelika Svetlove, Justus Alves, Alexander Kraupner, Francesca di Lillo, M. Andrea Markus, Giuliana Tromba, Frauke Alves, Christian Dullin

**Affiliations:** 1 Institute for Diagnostic and Interventional Radiology, University Medical Center Göttingen, Göttingen, Germany; 2 Translational Molecular Imaging, Max-Planck-Institute for Experimental Medicine, Göttingen, Germany; 3 nanoPET Pharma, Berlin, Germany; 4 Elettra Sincrotrone Trieste, Trieste, Italy; 5 Clinic for Hematology and Medical Oncology, University Medical Center Göttingen, Göttingen, Germany

**Keywords:** X-ray tomography, Image processing, Preclinical research

## Abstract

Although X-ray based 3D virtual histology is an emerging tool for the analysis of biological tissue, it falls short in terms of specificity when compared to conventional histology. Thus, the aim was to establish a novel approach that combines 3D information provided by microCT with high specificity that only (immuno-)histochemistry can offer. For this purpose, we developed a software frontend, which utilises an elastic transformation technique to accurately co-register various histological and immunohistochemical stainings with free propagation phase contrast synchrotron radiation microCT. We demonstrate that the precision of the overlay of both imaging modalities is significantly improved by performing our elastic registration workflow, as evidenced by calculation of the displacement index. To illustrate the need for an elastic co-registration approach we examined specimens from a mouse model of breast cancer with injected metal-based nanoparticles. Using the elastic transformation pipeline, we were able to co-localise the nanoparticles to specifically stained cells or tissue structures into their three-dimensional anatomical context. Additionally, we performed a semi-automated tissue structure and cell classification. This workflow provides new insights on histopathological analysis by combining CT specific three-dimensional information with cell/tissue specific information provided by classical histology.

## Introduction

Conventional histology of paraffin embedded tissue specimens has been the gold standard for the analysis of tissue samples for decades. While countless staining protocols and the use of microscopes allow very specific analysis down to (sub-)cellular level, histology falls short when it comes to acquiring 3D information of the tissue. Serial sectioning marginally circumvents this problem, but it is very time consuming, labour intensive and fails to deliver a real 3D dataset^1,2^. Therefore, techniques that can correlate cellular and morphological information within the 3D context of the entire tissue volume are in great demand.

MicroCT imaging can deliver high resolution 3D datasets^3^. Soft tissue specimens, however, require additional contrast commonly achieved using heavy ion-based staining methods. Our group, among others, has used different CT staining protocols to improve the specificity of microCT imaging. While phosphotungstic acid or iodine-based protocols can greatly increase the CT contrast of tissue, and therefore allow for a 3D assessment on a micrometer scale^4–8^, they do not increase the specificity to identify certain cells or tissue structures. Other approaches involve using reagents known from classical histological stainings. Eosin can be used to stain cytoplasm^9^, while hematein (led-III-acetate) functions as a nucleus specific staining agent^10,11^. While these approaches seem to take the specificity of microCT based virtual histology to a new level, they are hindered by limited sample sizes as well as by the fact that only a few applicable protocols exist to this day.

Metscher et al. showed that a specific antibody staining of chick embryos for microCT analysis is possible using silver labelled antibodies^12^. Due to the limited tissue penetration depth of the metal labelled antibodies this approach is limited to very small samples with relatively low tissue densities and can therefore not substitute classical immunohistochemistry.

However, staining can be entirely avoided by the use of free propagation-based phase contrast imaging, which can be performed using synchrotron light sources (SRµCT). This method has shown to be well suited deliver very detailed and three-dimensional information about the anatomical structure of the tissue samples and thereby depict the 3D architecture of unstained soft tissue specimens^6,13–17^. However, so far microCT cannot compete with histological approaches when it comes to the specific visualisation of cellular structures and tissues. Therefore, it would be of great interest to combine 3D microCT imaging and classical staining-based histology to take advantage of both imaging modalities.

In an earlier study we showed already that histological sections from paraffin embedded samples experience some non-uniform deformations during their preparation caused by the mechanical sectioning with a microtome, as well as the deparaffinisation and the staining procedure^7^. Similar findings were stated previously^18–20^. Furthermore, we demonstrated that this problem can be circumvented by using resin embedded samples. However, resin embedding also offers many disadvantages when compared to paraffin. The embedding itself takes much longer, the cutting requires special equipment, and the diversity of available staining methods is much smaller^21,22^. Thus, an improvement of the cross-method analysis of microCT and classical paraffin histology would benefit from an approach which compensates for the deformation caused by the histological processing is necessary.

One way to achieve this is to use image co-registration techniques. The typical approach entails one image that is kept unchanged and another image which is modified, optimising the registration quality. Modification can be simple translation and rotation if the object is considered to be rigid, otherwise affine and non-uniform transformations are also possible^23,24^. Optimising the parameters of the transformation requires a cost function which approaches its extreme when a perfect match is reached. Depending on the content of the two different images such a cost function can be cross-correlation or cross-entropy^7^ among others. Finally, an interpolation function is needed to apply the calculated transformation onto the second image. The entire approach is usually performed in a loop to iteratively optimise the parameters.

An illustrative example for an application of such a registration pipeline can be found in oncological nanomedicine. The depiction of nanoparticles (NPs) in their local tissue environment is an important matter for the characterisation of nanomaterial-based theranostics. As an example we used metal-based NPs to assess their radiotherapeutic enhancement in tumours, as has been explored before^25–27^. Achieving a homogenous distribution of NPs within the tumour is crucial. Optimisation of the NP delivery strategy, assessing their distribution and, more importantly, their correlation to necrotic areas and immune cells, is key. These parameters cannot be obtained through classical microscopy methods since NPs are usually below the resolution limit of optical microscopy and the tumours are too large for electron microscopy. At the same time, tumours possess many different types of tissue like the tumour tissue itself, fibrous areas, a tumour capsule and areas of different degrees of necrosis^28,29^, making them suitable for comparative histological analysis. In addition, this heterogeneous appearance increases the likelihood of non-uniform deformations during the sample preparation.

Using this model, we show that the combination of label-free 3D virtual histology and classical 2D paraffin-based histology can achieve a precise overlay of microCT and histology and/or immunohistochemistry by compensating for the unavoidable non-uniform deformations that occur during the sectioning and staining process. This enables the precise co-localisation of specifically stained cells or tissue structures into their three-dimensional anatomical context. As an example, we demonstrate that the approach can be implemented in the development of nanoparticle based therapeutic strategies by assessing the nanoparticle distribution in tumour tissue, which was not possible using a single imaging modality.

## Results

To develop the workflow of the co-registration between label free 3D microCT based virtual histology, classical histology and immunohistochemistry, we analysed tumour tissues from a mouse model of breast cancer by SRµCT and subsequent histological processing.

### Workflow for precise registration of histological sections and SRµCT data with Fuxlastix

**Figure 1:**
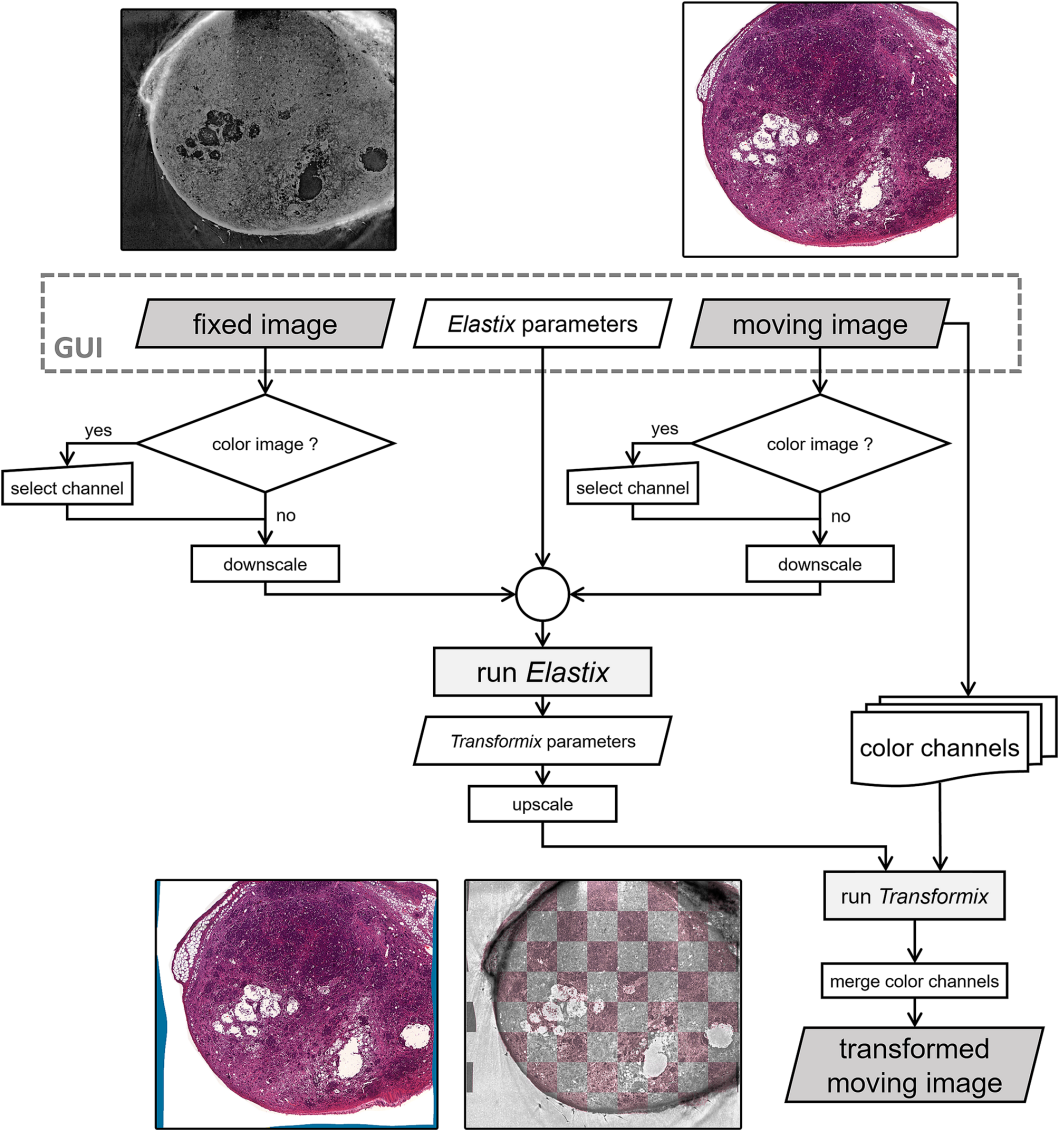
Image transformation pipeline using the Fuxlastix frontend. The fixed image (non-transformed CT image, top left), the moving image (histology image to be transformed, top right) and the Elastix parameter file are chosen manually in the user interface (GUI) of the software. If colour images are selected, the user needs to decide manually which colour channel should be used for the matching process. In addition, there is a choice to select the average of the colour channels as a grey value image and/or to invert the image. The images are downscaled by a factor of 10 and the Elastix software is called, which outputs the Transformix parameter file, which in turn is modified for the use on the original moving image. Each channel is transformed individually and subsequently merged to receive the final transformed moving image (bottom row, left image). The overlay of the fixed and the transformed overlay can be visualised using a checkerboard pattern (bottom row, right image).

Initial overlay of the CT and histological images required finding the position and orientation in the CT data set, which corresponds to the actual paraffin section, cut by the microtome. To achieve this, the CT data set was virtually cut with a plane that was manually adjusted until the virtual slice matched the histological slice. In order to compensate for the deformation due to cutting and staining procedures, a tailored pipeline was developed, as illustrated by the flowchart shown in Fig. 1. The virtual histology slice obtained from the 3D CT data set was used as a ‘fixed image’ template, representing the correct morphology before the histological processing. The histology slice was considered the ‘moving image’, representing non-uniform deformation upon the completion of histological processing. We then used a b-spline interpolation, which represents a grid that allows stretching and moving each position individually. Although useful for non-homogenous deformation, this model cannot handle strong global mismatches, especially rotations, as they are always approximated by deforming the grid. Therefore, initially the translation and rotation of the moving image with respect to the fixed image were corrected using a Fourier–Mellin algorithm^30,31^. For simplicity, either a single colour channel or the average of all channels from the moving image was used further. In addition, the histology images were inverted so the relevant image content is given as high grey values for both histology and CT. Both, the moving and the fixed images, were downscaled to 1/10th of their original size before registration with Elastix was performed. Mutual information^32^ was used as optimisation criteria and a b-spline as the deformation model^33^. The mutual information used as metric for registration is based on pairing the intensities at a certain pixel in both the fixed and moving image. To suppress the influence of noise, the intensity range was binned to 32 bins respectively. Respecting the scaling, the obtained deformation matrix was used on all colour channels of the moving image. The transformed channels were then merged into a single colour image. The resulting deformed image is shown in Fig. 1 (two bottom images). The blue border (Fig. 1, bottom left) indicates the degree of deformation calculated by the registration pipeline. The checkerboard view demonstrates the precision of the registration (Fig. 1, bottom right).

**Figure 2:**
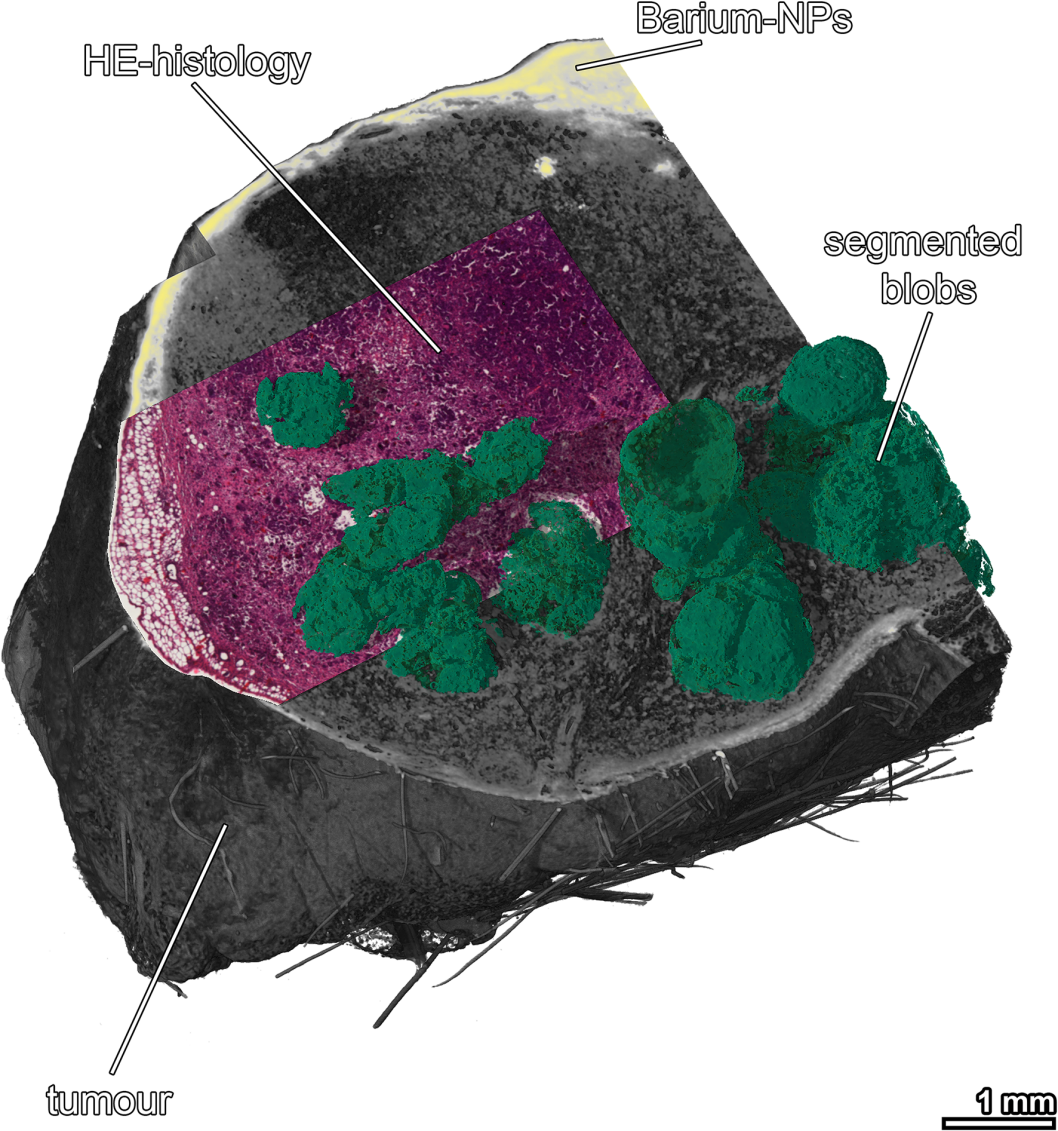
Capabilities of CT-based 3D virtual histology. 3D rendered mouse mamma tumour, virtually cut at the position of the matching HE histological section. Barium NPs are depicted in yellow. Necrotic "blobs" inside the tumour are shown in green. The "blobs" were segmented using a region growing algorithm, embedded in VG StudioMAX.

As an example, to demonstrate the capabilities of the pipeline, Fig. 2 illustrates a 3D rendering of a mouse breast tumour that was intratumourally injected with radiopaque barium nanoparticles (BaNPs). The data set was virtually cut open at the position of the subsequently performed histological section. The HE-stained section was registered to the virtual slice and placed as a plane into the 3D architecture of the tumour. The image demonstrates schematically that by using our workflow, specific information, like the occurrence of tumour cells or collagen fibres from the histology can be related to the 3D location within the entire specimen. The BaNPs, which are invisible in histology, can be easily discerned in the CT data set. In addition, 3D features like the position and extent of the “blobs”, which represent hollow cavities in the tumour and cannot be effectively analysed by histology, can be easily assessed volumetrically by CT.

### Evaluation of the registration quality

To evaluate if the deformation of the histology resulted in an optimal fit with the virtual cut through the CT data set, we calculated the previously introduced displacement index (DI)^8^. DI assesses the displacement and the mutual information using a block matching approach. An ideal match should result in a DI of 0. However, since histology and CT show different image content even for perfectly overlaid images, the DI will never reach this value.

**Figure 3:**
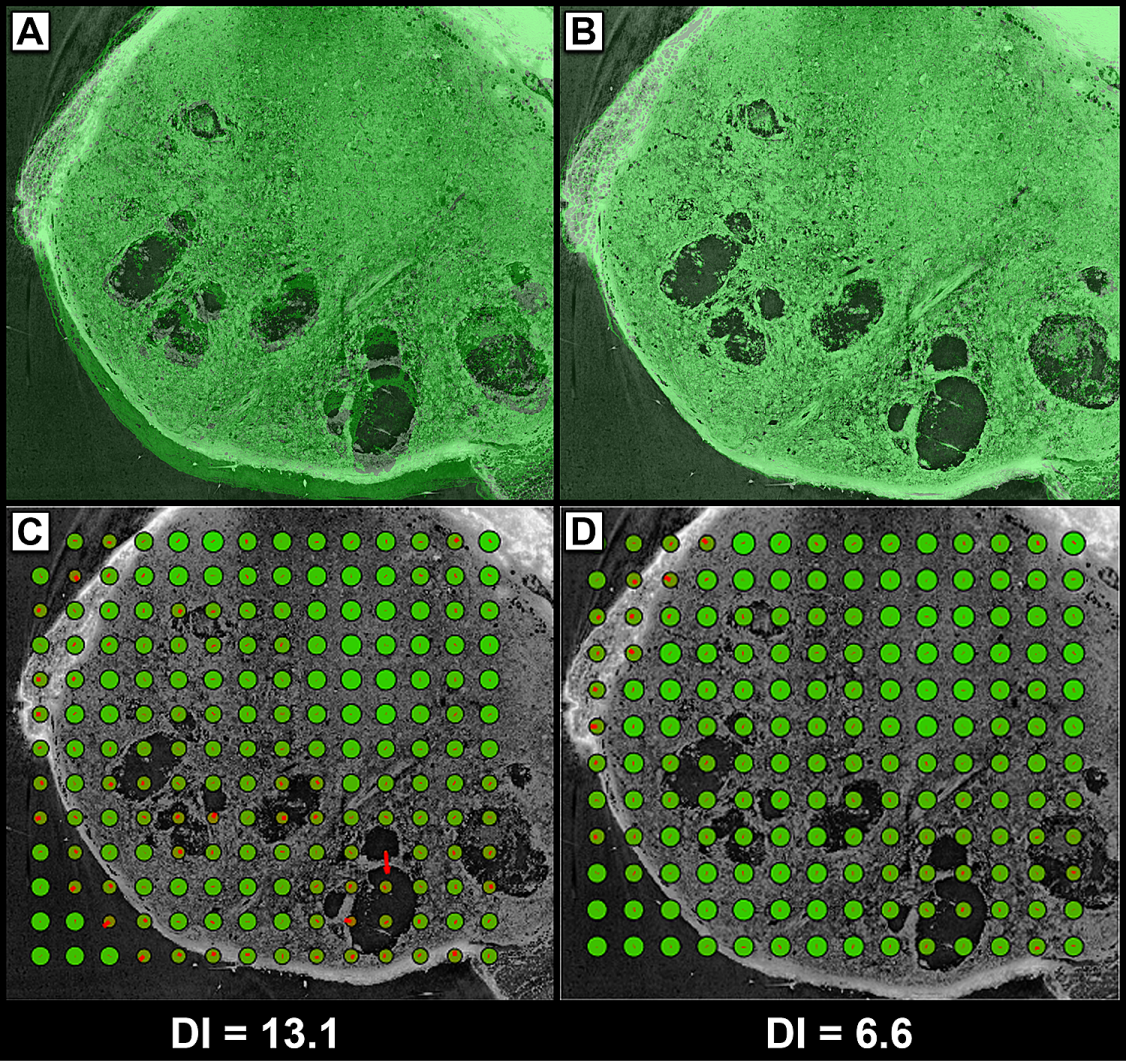
Evaluation of the registration quality using the ‘Displacement Index’. Representative CT (grey) and corresponding MTS (pseudo-coloured in green) images from a mouse breast cancer tissue. (**A**) Overlay of the CT image with the MTS histology image before transformation. (**B**) The same images after using the matching pipeline. (**C**) DI calculation of both images before matching (DI = 13.1). The congruence between each CT tile and histology tile is directly proportional to the size of the green circle inside that tile. The red lines show the calculated displacement of each tile. (**D**) DI calculation of both images after matching (DI = 6.6).

Figure 3A shows an overlay of CT data and the green colour channel of the corresponding MTS-stained histological slice (pseudo-coloured in green to improve visibility) before the registration. Evidently, the MTS data was deformed during the cutting and staining process and therefore did not match the CT slice. Figure 3B shows the same overlay after the histology data was transformed using Fuxlastix. It is clearly visible that a near perfect match was achieved. Figure 3C,D illustrate the calculation of the DI of the images pre- and post-transformation with a block size of 500 pixels with an overlap of 50%. The congruence between the CT tiles and the histology tiles is directly proportional to the size of the green circle inside that tile. The red lines show the calculated displacement of each tile. The results for all performed transformations demonstrate a larger DI of 11.7 ± 2.0 (n = 15) in the untransformed data as compared to a DI of 6.9 ± 2.0 (n = 15) in the transformed data sets, confirming a significant improvement of the overlay after registration of the histology data.

### Image registration of differently stained serial histological sections with microCT

**Figure 4:**
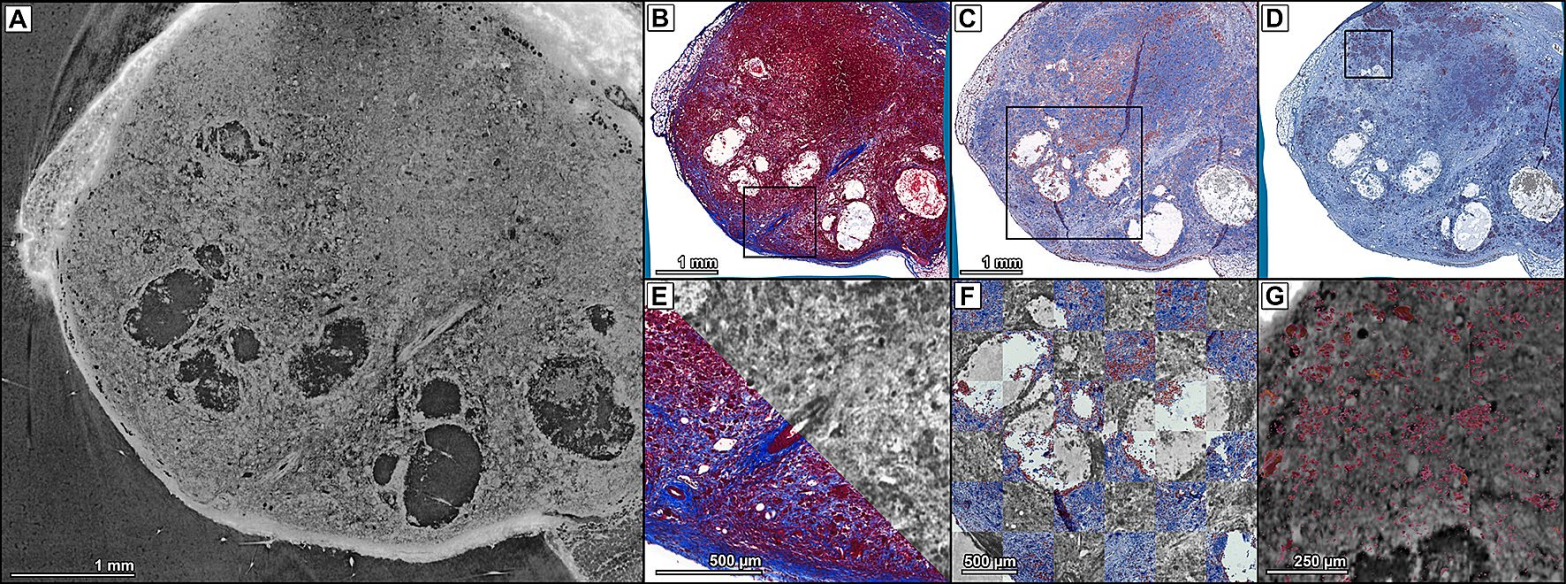
Matching of SRµCT data with different histological staining obtained from BaNPs injected mouse breast cancer tissues. (**A**) SRµCT virtual histology at the plane corresponding to the MTS histology shown in (**B**). (**B**) MTS histology. (**C**) Anti-CD68 immunohistochemistry. (**D**) Anti-TAg immunohistochemistry. (**E**) Comparison of MTS staining with the corresponding CT slice of a magnified region as indicated by the black box in (**B**). Grey values are inverted. (**F**) Checkerboard comparison of anti-CD68-IHC staining with the corresponding CT slice of a magnified region as indicated by the black box in (**C**). Grey values are inverted. (**G**) Overlay of segmented T-Ag positive tumour cells with the corresponding CT slice of a magnified region as indicated by the black box in (**D**).

In order to provide a multi-parametric analysis of tissue samples, different staining protocols are commonly applied on consecutive histological sections. Since every histological slice may display different degrees of non-uniform deformation, a direct overlay provides limited information. To correct for various degrees of deformation between the different histological slices, we used an unaltered CT data set as a fixed image template. We applied our elastic image registration technique on every corresponding virtual cut through the CT data set of mammary tumours (Fig. 4A) and performed separate registration with consecutive slices stained with different techniques. Masson Trichrome staining (MTS, Fig. 4B) was applied to depict collagen fibres in blue. Figure 4E shows a magnification and explicitly demonstrates the continuity of the structures achieved by the registration. Specific structures like the blood vessel were identified through histology and confirmed by tracking the vessel throughout the 3D CT data set. Figure 4C,D show specific IHC stainings for the macrophage and monocyte marker CD68 and for the SV40 T-Antigen (T-Ag), used as specific tumour marker in the WAP-T tumour mouse model, matched to their corresponding virtual slice. CD68 and T-antigen positive cells are depicted in red. Detailed views of the localisation of IHC positive stained cells are visualised by a checkerboard view for the anti-CD68 IHC (Fig. 4F) and an alpha blending overlay of the segmented T-Ag positive tumour cells (Fig. 4G). These findings demonstrate that the proposed workflow can be successfully applied for co-registration of microCT and histology/IHC data independently of the staining type. In addition, individual corrections of non-uniform deformations for every histological slice allow for effective comparison of the different staining techniques by removing the deformation-based variability between the different serial sections.

This approach enables the selective comparison of cells and structures within specific regions in the different sections, which would not be possible without the co-registration.

### Allocation of NP accumulation sites to anatomical structures in mouse breast tumour samples

**Figure 5:**
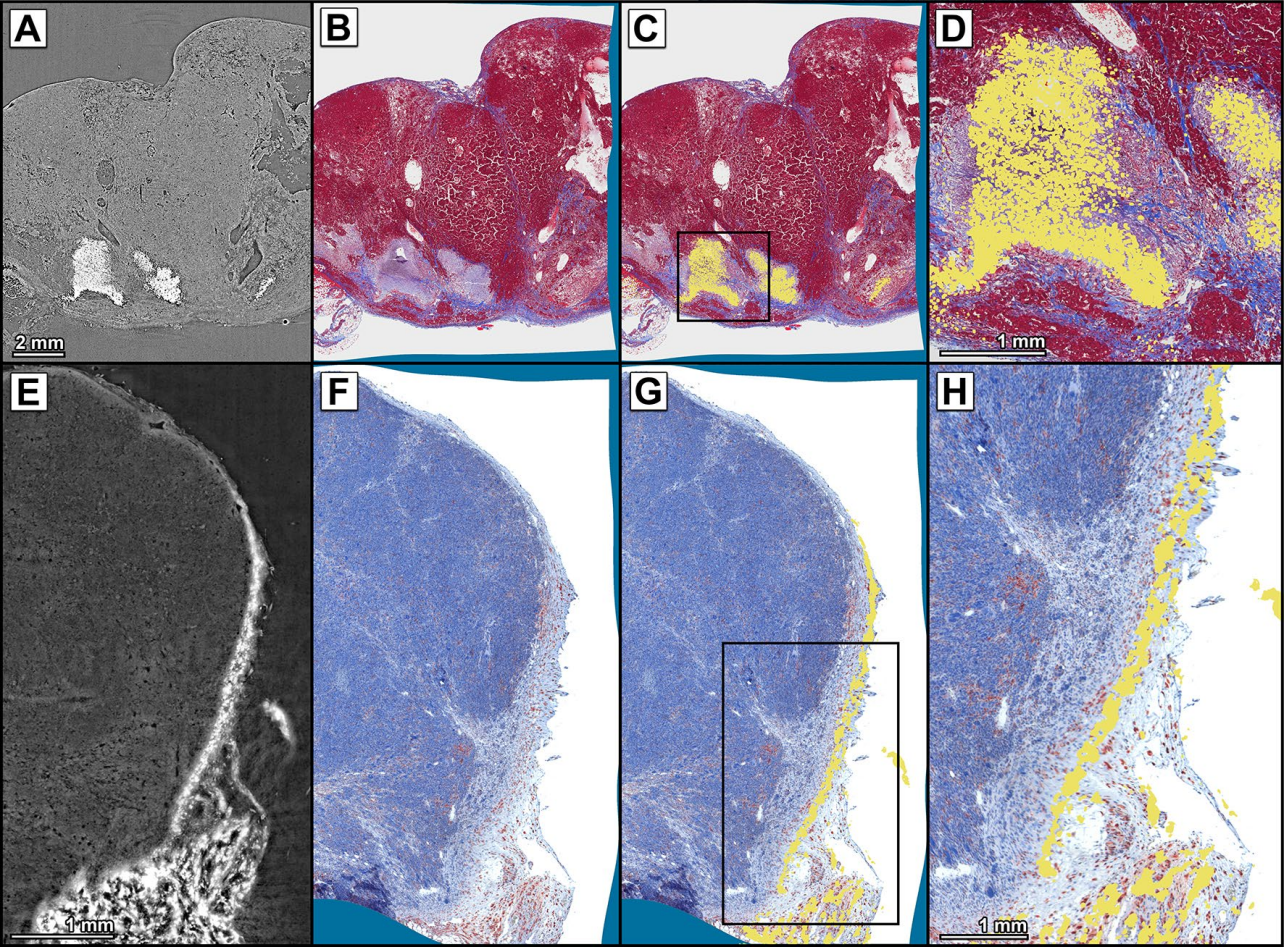
Matching of SRµCT datasets with classical histology allows precise localization of BaNPs in histological slices. (**A**) SRµCT of a mouse breast tumour. Injected BaNPs can be easily localised within the tumour tissue, due to their high CT contrast. (**B**) Registered MTS-stained histological slice of the tumour in (**A**). Deformation is indicated by the light blue border. (**C**) Mage fusion of the MTS staining shown in (**B**) with the segmented BaNPs from (**A**). BaNPs were segmented facilitating a threshold-based approach using Otsu’s Method. (**D**) Magnification of the region indicated by the black box in (**C**). (**E**) SRµCT slice of a second mouse breast tumour. BaNPs are localized in the tumour capsule and in the adjacent fatty tissue. (**F**) Registered anti-CD68 IHC slice of the tumour in (**E**). (**G**) Image fusion of the anti-CD68 IHC image in (**F**) with segmented BaNPs from (**E**). BaNPS are located at the same site as CD68 positive cells indicating an uptake of the BaNPs by these phagocytic cells. (**H**) Magnification of the region indicated by the black box in (**G**).

In order to evaluate our workflow in the scope of a biomedical question, we applied the developed registration workflow in a NP based theranostic strategy to assess NP distribution as one of the key issues. BaSO_4_ containing NPs were injected directly into the mouse breast tumours, followed by low energy irradiation over multiple therapy sessions. The tumours were then explanted, and high-resolution CT and histology were performed as described. The high image quality of the embedded breast tumour in CT was achieved by applying propagation-based imaging without the use of additional contrast agents, which would have reduced the relative contrast of the nanoparticles. BaNPs were easily discerned (Fig. 5A), due to their high X-ray contrast. The BaNPs, however, could not be visualised in histology as shown in the corresponding MTS-stained slice (Fig. 5B). Thus, we applied the same workflow described above in order to place the BaNPs in the anatomical context of the tumour. Figure 5B shows the histological slice corrected for deformations during the cutting process as indicated by the inhomogeneous boundary regions coloured in blue. Figure 5C shows the same histological slice with the overlaid and segmented BaNPs. The BaNPs were segmented by using a simple thresholding method (Otsu’s method) and are shown by a yellow pseudo-colour enabling to depict the position of NPs within the tumour.

In the tumour shown here, BaNPs are concentrated in the periphery of necrotic regions, which are characterised by the lack of red staining in MTS. As indicated in the close-up image (Fig. 5D), our registration approach allows to analyse the local environment of small clusters of particles. Since the NPs were designed for contrast enhanced radiation therapy, we assume that the visible necrotic regions were most likely formed due to the increased local radiation damage caused by the particles.

The co-registration shown in Fig. 5E depicts another example of a breast tumour where the BaNPs can be allocated only to the tumour capsule, indicating a different pattern of NP distribution after intratumoural injection. Figure 5F illustrates the corresponding anti-CD68 stained IHC slice after the registration was performed. Furthermore, the fusion with an anti-CD68 IHC stained slice (Fig. 5G) demonstrates that the localisation of the NPs coincides with regions of CD68-positive cells, indicative of macrophages that have phagocyted the BaNPs. Figure 5H shows a magnified detail view of Fig. 5G clearly depicting a co-localisation of the BaNPs and phagocytic cells.

These examples neatly demonstrate how the information unachievable by classical histological analysis can be supplemented by means of registration with high resolution CT.

### Precise co-registration of microCT and histology improves automatic cell classification

**Figure 6:**
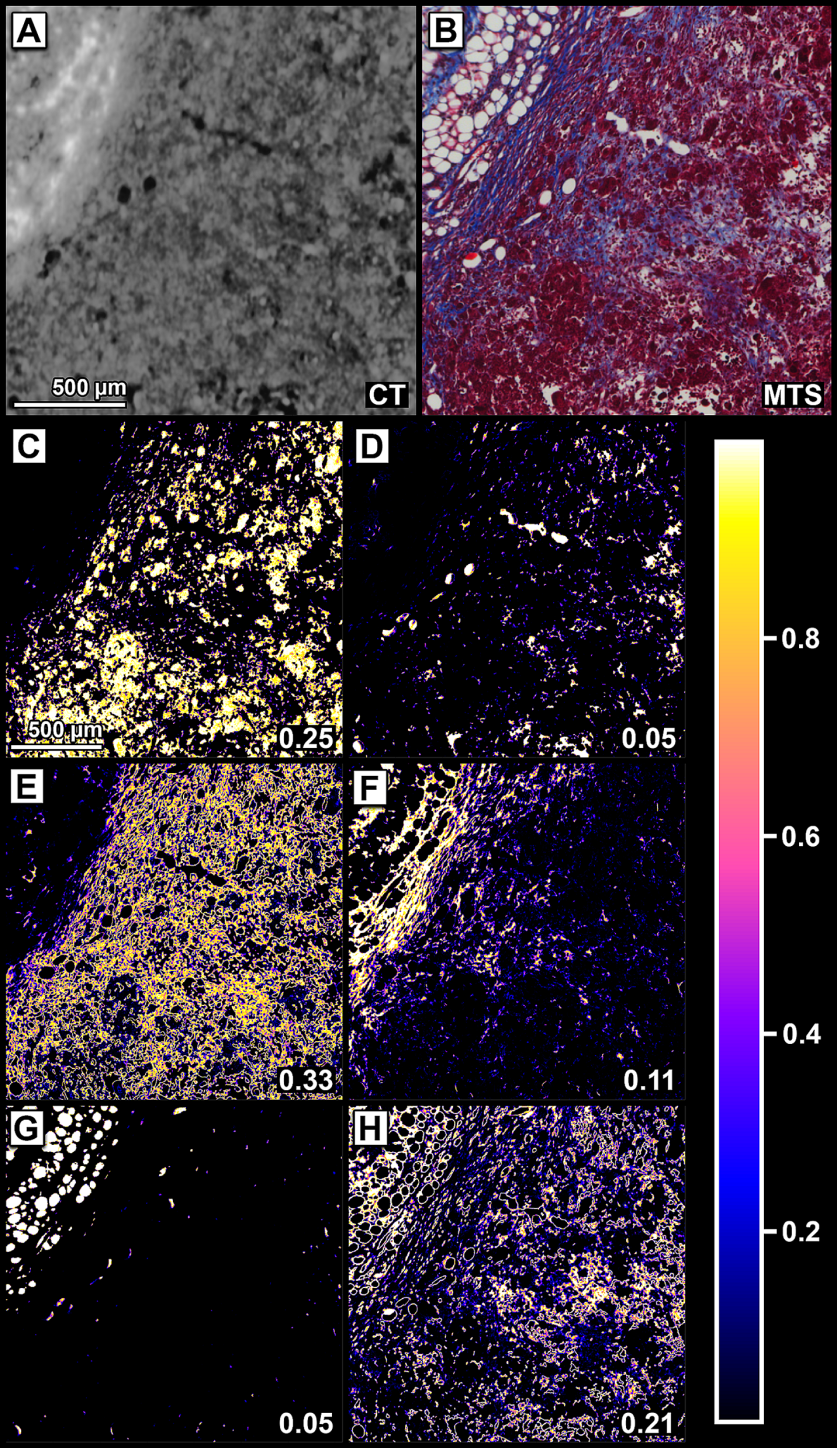
Automatic classification of tissue structures using registered CT and histology. (**A**) CT image. (**B**) MTS histology image matched to the CT image shown in (**A**). (**C**–**H**) Colour coded probability maps of the six different components of the gaussian mixed model optimized by EM. The colour code for the different probabilities can be seen in the bar. (**C**) Mainly depicts healthy tumour cells. (**D**) Depicts vessels and holes within the tissue. (**E**) Shows NP location, extracellular matrix components. (**F**) Depicts the BaNPs. (**G**) Displays fat cells. (**H**) Displays cell membranes. All images (**C**–**H**) also state the mix coefficient in the lower right corner. Fibres components (**E**) are the most dominant component with a mix coefficient of 35% followed by the healthy tumour cells with 25%.

As an example of how combined analysis of microCT and histology can be used to improve automatic cell or tissue classification, we used a pixel-wise mixed density model approach. Figure 6 shows a microCT image of a local area of a mammary tumour containing BaNPs (Fig. 6A) and the corresponding part of the MTS-stained histological slice elastically matched using the proposed pipeline (Fig. 6B). Thus, each pixel is characterised by its intensity value of the CT data and its three colour components of the MTS staining generating a 4D feature space. Since it cannot be expected that the registration between the two images is precise on a pixel-wise basis, the feature space showed highly overlapping clusters limiting the automatic classification of different structures. Therefore, we used a 4D Gaussian mixed model with 6 components that was automatically adapted to the data by applying an expectation maximisation algorithm implemented in ski-learn. Consequently, the probability for each pixel to belong to each of the 6 classes is shown in Fig. 6C–H. As a result, the class shown in Fig. 6C represents the tumour cells (25% probability). Extra cellular matrix components, such as collagen are represented by the class shown in Fig. 6E. The fat cells (Fig. 6G) can easily be separated from small vessels and empty areas in the tissue (Fig. 6D), which would not have been possible using only the histological slice (Fig. 6B). Since the fat contained in the adipocytes is washed out during the staining process, the fat cells appear to be indistinguishable from the porous morphology of the tissue, making the ability to segment them even more remarkable. Figure 6F mainly depicts the blue stained fibre content in the MTS staining, while Fig. 6H highlights interfaces and cell membranes between the different cell types. Our results show that a simple tissue classification can be improved by using correlative CT and histology data sets. Without the combined information provided by the CT and the registered MTS histology an automatic classification into the shown classes was not successful, as shown in the supplemental files (Figure S1, S2).

## Discussion

Here we present a novel workflow to precisely register histological sections with virtual cross-sections within microCT data sets of paraffin embedded tissue and demonstrate its performance on tumours of a breast cancer mouse model.

We developed a simple to use workflow based on Elastix^34,35^ to perform an elastic registration of the data sets, which is available here https://github.com/xPITcoding/Fuxlastix. We show that a near cellular precision between two imaging modalities can be achieved. To simplify the registration, we reduced a 2D–3D problem to a 2D–2D problem by performing a manual virtual slicing of the CT data. While this makes the registration itself much easier, the resulting quality of the overlay is always limited by the precision of the initial virtual sectioning. Even though we believe that an automated co-registration technique would not necessarily offer a more precise match of both modalities, it could speed up the whole workflow significantly. Since cutting of the histological sections is performed by a microtome, which is essentially a static blade, it is reasonable to assume that the histological slice can be described by a 2D plane through the microCT data set and the deformation associated with the cutting is restricted to that plane.

In the literature most methods for image co-registration are either intensity, landmark or segmentation based^36–39^. Since, in our case, microCT data sets and histology have a vastly different image content a flexible registration method was needed. In addition, both SRµCT and histology are prone to artifacts (air bubbles, paraffin cracks, tissue breakage, uneven staining). Therefore, a very robust registration method was required. Because of this, we used a mutual information-based registration approach, rather than a landmark based method. Chicherova et al. demonstrated successful registration between microCT and histology in 3D using a SURF (Speeded Up Robust Features) algorithm^40^. Since SURF is based on the automatic identification of corresponding landmarks, it appears to work well on histochemical stainings such as the HE staining presented in their publication. In the case of antibody-based staining, in which the image content differs strongly between CT and histology, we speculate that the application of SURF would be challenging. In contrast to SURF’s landmarking, our approach uses mutual information and considers the entire image content, which has proven to be more robust and precise in our IHC data. However, Chicherova et al. achieved the best results when a curved plane was fit to the 3D data set, while our approach works under the assumption of a flat plane and 2D registration. Therefore, it is reasonable to further evaluate whether the registration quality can be improved by forfeiting the assumption that the microtome cuts strictly on a plane. Additionally, Chicherova et al. settled on a deformation model based on Legendre-polynomials in order to avoid the flexibility of the b-splines^40^. This is indeed a reasonable approach when applied in dense tissues like bone and cerebellum. However, our specimens displayed a large variability in deformation magnitude at different regions in the tissue. Thus, we believe that the b-spline approach was indeed required in our case to achieve a precise match. On the other hand, we are aware that our elastic registration pipeline cannot be perfectly validated as no ground truth data of an optimal overlay can be generated and elastic image registration will always converge to a solution. In the future, with the availability of more data, the maximal local deformations for a given tissue can be evaluated and used to modify the penalty term to a solution with large local deformations of the b-splines.

It is important to point out, that SRµCT does not interfere with subsequent staining procedures of paraffinized samples commonly employed in clinical routine. SRµCT can deposit very large X-ray doses in the sample (in the range of kGy for the samples shown here). In this study we did not apply any additional staining to increase the X-ray contrast of the sample, therefore the absorption is relatively low, leading to reduced dose deposition. In contrast to that, our former experiments^6^ using phosphotungstic acid (PTA) stained tissue samples led to a significantly increased X-ray absorption and therefore dose deposition. SRµCT in PTA stained samples can lead to micro cracks in the paraffin, which can impede the sectioning process. For the samples presented here, no visible crack formation was observed. For both PTA stained and unstained SRµCT scanned specimens no significant impairment of histology and IHC was observed. Paraffin blocks, when compared to other embedding materials, provide a number of advantages such as: (i) access to more staining protocols, (ii) easier handling and (iii) use of subsequent analysis techniques such as atomic force microscopy. Thus, our approach may be integrated into the standard workflow of histopathology.

In BaNP injected mouse tumours we demonstrated that our approach facilitates the correlation of NP location to anatomical features such as fibre structures as components of the extracellular matrix, cell density in necrotic areas and macrophage localisation. However, since histological sections are performed in a serial manner, they do not represent the same cells/tissue structures. The co-localisation to virtual histology slices can therefore not result in an optimal match on cellular level for every slice. New approaches offer the advantage of having multiple antibody stainings on the same slice^41,42^. This would enable an improved multiplexing of microCT data with information from multiple stainings. Therefore, artificial staining of the microCT 3D dataset by using machine learning approaches to transfer the appearance (style) of histology to the microCT data is feasible, even at locations for which a corresponding histological slice does not exist. A similar approach based on style transfer learning has been established for unstained tissue images, where artificial HE and MTS stainings were performed based on the intensity of autofluorescence of the tissue^43^. We are thus confident that a pseudo-histological-stained X-ray based virtual 3D histology could be achieved and would furthermore allow to perform histology evaluation at any position and orientation in no time. Moreover, the specimen will not be destroyed and multiple virtual stainings would be possible.

Since our approach allows a precise match between histology and virtual microCT sections, all information can be assigned to the same structure at near cellular level. Such a location can be described by a multi-dimensional feature vector as demonstrated by the combination of the local grey values in the microCT data with the intensities of the three different colour channels in the MTS-stained slice. These feature vectors enable us to employ unsupervised machine learning techniques to identify different cell and/or tissue types. We demonstrated this by the automated separation into healthy tumour regions, fibre regions, fat cells and NP positive regions. The combination of more and different stainings will help to further improve this technique.

In summary, we believe that this workflow based on our open-source frontend for Elastix could aid the histological tissue analysis by supplying additional 3D information, in a large variety of applications in biomedical research. Moreover, it might present a valuable starting point for machine learning strategies and a deeper understanding of pathological processes.

## Methods

### NanoPET BaSO_4_ nanoparticles

Barium sulphate based nanoparticles (BaNPs) were produced by chemical precipitation at ambient conditions^44^. After a purification step, the particles were sterically stabilised using a biocompatible polymer. The obtained highly stable colloidal suspension was sterilised and formulated for in vitro/in vivo use. The hydrodynamic diameter of the particles was around 120 nm.

### Animal model

Breast tumour cells (pH8N8) derived from tumours arising in bi-transgenic SV40 T-antigen/mutant p53 (WAP-T/WAP-mutp53) mice were maintained and cultured as described before^28,45–47^. 1 × 10^6^ tumour cells were orthotopically implanted into syngeneic female WAP-T NP8 mice as described^29^.

When the tumours reached a size of 300 mm^3^, 15 µl of BaNPs were injected intratumourally by performing three injections at different sites with a 10 µl glass syringe (Hamilton), followed by irradiation of the tumours with a total dose of 30 Gy over 15 therapy sessions in a small animal in vivo microCT (QuantumFX, Perkin Elmer).

The mice were sacrificed using a CO_2_ overdose in combination with a cervical dislocation. The tumours were explanted, fixed in 4% formalin, and embedded in paraffin for further histological analysis.

All animal in-vivo procedures were performed in compliance with the guidelines of the European Directive (2010/63/EU) and the German animal ethics regulations and were approved by the local ethics office (Niedersächsisches Landesamt für Verbraucherschutz und Lebensmittelsicherheit, LAVES, ethics approval 33.9-42502-04-18/3022. Furthermore, the study was carried out in compliance with the ARRIVE guidelines (https://arriveguidelines.org/).

### Synchrotron radiation microCT (SRµCT)

All high-resolution SRµCT acquisitions were performed using the white/pink beam setup of the SYRMEP (SYnchrotron Radiation for MEdical Physics) beamline of the Italian synchrotron ELETTRA (Trieste, Italy).

Whole paraffin embedded samples were scanned with a sample to detector distance of 500 mm. A a 16-bit, water-cooled sCMOS camera (Hamamatsu C11440-22C ORCA-Flash 4.0 v2) was used to acquire 3600 angular distributed projections with an exposure time of 50 ms and an isotropic voxel size of 2.33 µm. Scans were performed in a 360° offset regime, resulting in a scan time of 180 s. A 0.5 mm Si filter was used, resulting in a mean beam energy of 19.6 keV. All SRµCT data sets were reconstructed using SYRMEP Tomo Project^48^ software (STP Version 1.3.2). A single distance phase retrieval algorithm developed by Paganin et al.^49^ was used with a delta over beta ratio of 100. To image the entire tumours, 3–4 single acquisitions were performed, and the resulting reconstructions were manually stitched together using Fiji (NIH).

### Histology and immunohistochemistry (IHC)

Paraffin-embedded samples were sectioned in slices of 2 µm thickness using a standard microtome (HM 340 E microtome, Thermo Fischer Scientific). Histological haematoxylin and eosin (HE) and Masson trichrome staining (MTS), as well as immunostaining of SV40 T-antigen (T-Ag) marker and the macrophage specific marker CD68 were performed as described previously^29^. In brief, a Masson trichrome kit (MTS, Merck) was applied to stain collagen. A polyclonal rabbit anti-mouse CD68 antibody (abcam 125212, 1:500) was used to stain macrophages and monocytes. Since H8N8 breast tumour cells express T-Antigen, this protein was used as a marker for tumor cells and specifically stained with anti-SV40 T-Antigen antibody (homemade rabbit antibody, 1:5000)^50^.

Histological images were acquired using an Axiovert 200 inverted microscope (Zeiss). To depict the whole tumour sections, the built-in stitching algorithm of the microscope was used with a 10 × magnification.

### Elastic registration of microCT and histological images

Resampling of the CT dataset and identification of the appropriate matching virtual slice was done using VG Studio MAX (V3.1, Volume Graphics). Fiji was used to pre-process the histological images. Calculation of the deformation and transformation of the images was done via Elastix and Transformix^34,35^ that were integrated into a tailored graphic user interface called Fuxlastix. The Fuxlastix frontend was developed in C++, using QT5.15 and is available on https://github.com/xPITcoding/Fuxlastix. For the presented data, the registration process took less than a minute using a standard computer with a quad-core CPU.

### Quantification of registration accuracy

The images acquired by CT and histology were compared in a block matching technique, calculating the mutual information MI and the displacement vector d for each block *i*. DI is then calculated as

$${\text{DI}} = \frac{1}{G}\mathop \sum \limits_{i}^{{}} \overline{{d_{i} }} MI_{i}^{ - 1}$$

with $G = \sum\nolimits_{i} {MI_{i}^{ - 1} }$ and $\overline{{d_{i} }}$ the length of the displacement vector, as previously described^7^. Images with a high similarity are therefore characterised by a minimal DI. Fifteen tumour sections stained with histological staining protocols were evaluated. Statistical significance was determined using an unpaired, two-tailed Student's t-test. A p-value of 0.05 or lower was considered statistically significant.

### Tissue classification using mixed density models

Classification of different structures in the combined data of histology and microCT was based on the assumption that after successful registration each pixel is characterised by four values—the three colour components of the histology image and the grey value of the microCT data set. Thus, similar tissue/cell types are characterised by similar combinations of those values. Classification was then performed by cluster analysis. Because the feature space is strongly overlapping due to partial volume effects and limitation of the registration approach, we used a mixed model of Gaussian distributions^51^. The parameters of these distributions were then evaluated by an expectation maximisation algorithm (EM) resulting in probabilities for each pixel to belong to a certain component/class of this model. We used the EM implementation of the python module scikit-learn^52^.

## Supplementary Information

Supplementary Figures.

## References

[1] Zankel A, Wagner J, Poelt P (2014). Serial sectioning methods for 3D investigations in materials science. Micron.

[2] Pichat J, Iglesias JE, Yousry T, Ourselin S, Modat M (2018). A survey of methods for 3D histology reconstruction. Med. Image Anal..

[3] Albers J (2018). X-ray-based 3D virtual histology—Adding the next dimension to histological analysis. Mol. Imaging Biol..

[4] Dullin C (2017). μCT of ex-vivo stained mouse hearts and embryos enables a precise match between 3D virtual histology, classical histology and immunochemistry. PLoS ONE.

[5] Metscher BD (2009). MicroCT for developmental biology: A versatile tool for high-contrast 3D imaging at histological resolutions. Dev. Dyn..

[6] Saccomano M (2018). Synchrotron inline phase contrast µCT enables detailed virtual histology of embedded soft-tissue samples with and without staining. J. Synchrotron Radiat..

[7] Albers J, Markus MA, Alves F, Dullin C (2018). X-ray based virtual histology allows guided sectioning of heavy ion stained murine lungs for histological analysis. Sci. Rep..

[8] Metscher BD (2009). MicroCT for comparative morphology: Simple staining methods allow high-contrast 3D imaging of diverse non-mineralized animal tissues. BMC Physiol..

[9] Busse M (2018). Three-dimensional virtual histology enabled through cytoplasm-specific X-ray stain for microscopic and nanoscopic computed tomography. Proc. Natl. Acad. Sci..

[10] Metscher B (2021). A simple nuclear contrast staining method for microCT-based 3D histology using lead(II) acetate. J. Anat..

[11] Müller M (2018). Nucleus-specific X-ray stain for 3D virtual histology. Sci. Rep..

[12] Metscher BD, Müller GB (2011). MicroCT for molecular imaging: Quantitative visualization of complete three-dimensional distributions of gene products in embryonic limbs. Dev. Dyn..

[13] Frohn J (2020). 3D virtual histology of human pancreatic tissue by multiscale phase-contrast X-ray tomography. J. Synchrotron Radiat..

[14] Kitchen MJ (2017). CT dose reduction factors in the thousands using X-ray phase contrast. Sci. Rep..

[15] Mohammadi S (2014). Quantitative evaluation of a single-distance phase-retrieval method applied on in-line phase-contrast images of a mouse lung. J. Synchrotron Radiat..

[16] Dullin C (2021). Multiscale biomedical imaging at the SYRMEP beamline of Elettra—Closing the gap between preclinical research and patient applications. Phys. Open.

[17] Kommoss FK (2021). Three-dimensional virtual histology of benign and malignant endometrial stromal neoplasms: A new perspective on why morphology matters. Int. J. Gynecol. Cancer.

[18] Gibson E (2013). 3D prostate histology image reconstruction: Quantifying the impact of tissue deformation and histology section location. J. Pathol. Inform..

[19] Alyami W, Kyme A, Bourne R (2020). Histological validation of MRI: A review of challenges in registration of imaging and whole-mount histopathology. J. Magn. Reson. Imaging.

[20] Samavati N (2011). Biomechanical model-based deformable registration of MRI and histopathology for clinical prostatectomy. J. Pathol. Inform..

[21] Eastham WN, Essex WB (1969). Use of tissues embedded in epoxy resin for routine histological examination of renal biopsies. J. Clin. Pathol..

[22] Johnson G, Zhang M, Barnett R (2000). A comparison between epoxy resin slices and histology sections in the study of spinal connective tissue structure. J. Int. Soc. Plastination.

[23] Oliveira FPM, Tavares JMRS (2014). Medical image registration: A review. Comput. Methods Biomech. Biomed. Eng..

[24] Zitová B, Flusser J (2003). Image registration methods: A survey. Image Vis. Comput..

[25] Chen Q (2019). Nanoparticle-enhanced radiotherapy to trigger robust cancer immunotherapy. Adv. Mater..

[26] Hainfeld JF, Slatkin DN, Smilowitz HM (2004). The use of gold nanoparticles to enhance radiotherapy in mice. Phys. Med. Biol..

[27] Jain S, Hirst DG, O’Sullivan JM (2012). Gold nanoparticles as novel agents for cancer therapy. Br. J. Radiol..

[28] Maenz C (2015). Epithelial–mesenchymal plasticity is a decisive feature for the metastatic outgrowth of disseminated WAP-T mouse mammary carcinoma cells. BMC Cancer.

[29] Jannasch K (2015). Chemotherapy of WAP-T mouse mammary carcinomas aggravates tumor phenotype and enhances tumor cell dissemination. Int. J. Cancer.

[30] Dasgupta B, Chatterji BN (1996). Fourier–Mellin transform based image matching algorithm. IETE J. Res..

[31] Guo, X., Xu, Z., Lu, Y. & Pang, Y. An application of Fourier–Mellin transform in image registration. in *The Fifth International Conference on Computer and Information Technology *(*CIT’05*) 619–623 (2005). 10.1109/CIT.2005.62.

[32] Thévenaz P, Bierlaire M, Unser M (2008). Halton Sampling for Image Registration Based on Mutual Information. Sampl. Theory Signal Process. Data Anal..

[33] Rueckert D (1999). Nonrigid registration using free-form deformations: Application to breast MR images. IEEE Trans. Med. Imaging.

[34] Klein S, Staring M, Murphy K, Viergever MA, Pluim JPW (2010). elastix: A toolbox for intensity-based medical image registration. IEEE Trans. Med. Imaging.

[35] Shamonin DP (2014). Fast parallel image registration on CPU and GPU for diagnostic classification of Alzheimer’s disease. Front. Neuroinformatics.

[36] Ohnishi T (2016). Deformable image registration between pathological images and MR image via an optical macro image. Pathol. Res. Pract..

[37] Wodzinski M, Skalski A (2020). Multistep, automatic and nonrigid image registration method for histology samples acquired using multiple stains. Phys. Med. Biol..

[38] Wodzinski M, Müller H (2021). DeepHistReg: Unsupervised deep learning registration framework for differently stained histology samples. Comput. Methods Programs Biomed..

[39] Borovec, J., Munoz-Barrutia, A. & Kybic, J. Benchmarking of image registration methods for differently stained histological slides. in *2018 25th IEEE International Conference on Image Processing *(*ICIP*) 3368–3372. 10.1109/ICIP.2018.8451040 (2018).

[40] Chicherova N (2018). Automatic deformable registration of histological slides to μCT volume data. J. Microsc..

[41] Pankratz, J. *et al.* Iterative ultrahigh-content imaging with the MACSima™ Imaging Platform using novel releasable antibody-fluorochrome conjugates based on REAlease^®^ Technology, 1. https://www.miltenyibiotec.com/_Resources/Persistent/1b329dcf7d3010e232862055597b0a2722f81609/Pankratz_CYTO_2019.pdf.

[42] Reiß S (2019). Characterization and classification of glioblastoma multiforme using the novel multiparametric cyclic immunofluorescence analysis system MACSima. Cancer Res..

[43] Rivenson Y (2019). Virtual histological staining of unlabelled tissue-autofluorescence images via deep learning. Nat. Biomed. Eng..

[44] Ramos-Gomes F, Ferreira N, Kraupner A, Alves F, Markus MA (2020). Ex vivo live cell imaging of nanoparticle-cell interactions in the mouse lung. Front. Bioeng. Biotechnol..

[45] Lenfert E (2015). Mutant p53 promotes epithelial–mesenchymal plasticity and enhances metastasis in mammary carcinomas of WAP-T mice. Int. J. Cancer.

[46] Schulze-Garg C, Löhler J, Gocht A, Deppert W (2000). A transgenic mouse model for the ductal carcinoma in situ (DCIS) of the mammary gland. Oncogene.

[47] Krepulat F (2005). Epigenetic mechanisms affect mutant p53 transgene expression in WAP-mutp53 transgenic mice. Oncogene.

[48] Brun F (2017). SYRMEP Tomo Project: A graphical user interface for customizing CT reconstruction workflows. Adv. Struct. Chem. Imaging.

[49] Paganin D, Mayo SC, Gureyev TE, Miller PR, Wilkins SW (2002). Simultaneous phase and amplitude extraction from a single defocused image of a homogeneous object. J. Microsc..

[50] Wegwitz F (2010). Tumorigenic WAP-T mouse mammary carcinoma cells: A model for a self-reproducing homeostatic cancer cell system. PLoS ONE.

[51] Redner RA, Walker HF (1984). Mixture densities, maximum likelihood and the Em algorithm. SIAM Rev..

[52] Pedregosa, F. *et al.* Scikit-learn: Machine Learning in Python. *Mach. Learn. PYTHON* 6.

