# Supplementary material for: Elastic transformation of histological slices allows precise co-registration with microCT data sets for a refined virtual histology approach
Source: Sci Rep. 2021 May 25;11:10846. doi: 10.1038/s41598-021-89841-w (PMC8149420; doi:10.1038/s41598-021-89841-w)
Supplement: Supplementary file 1 — Supplementary Figures. [file 41598_2021_89841_MOESM1_ESM.docx]

**Elastic transformation of histological slices allows precise co-registration with microCT data sets for a refined virtual histology approach**

Jonas Albers^1*^, Angelika Svetlove^1,2^, Justus Alves^1^, Alexander Kraupner^3^, Francesca di Lillo^4^, M. Andrea Markus^2^, Giuliana Tromba^4^, Frauke Alves^1,2,5^ and Christian Dullin^1^

1. Institute for Diagnostic and Interventional Radiology, University Medical Center Goettingen, Goettingen, Germany
2. Translational Molecular Imaging, Max-Planck-Institute for Experimental Medicine, Goettingen, Germany
3. nanoPET Pharma, Berlin, Germany
4. Elettra Sincrotrone Trieste, Trieste, Italy
5. Clinic for Hematology and Medical Oncology, University Medical Center Goettingen, Goettingen, Germany

**Supplemental Files:**


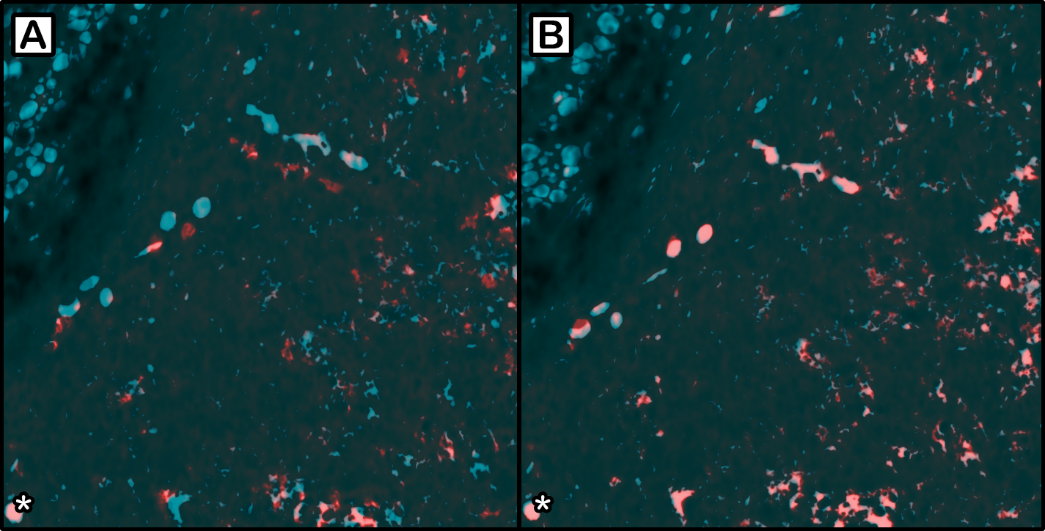


*Figure S1***: *Overlay of input images for the performed tissue classification as shown in Figure 6.***

A: Overlay of microCT (red) and MTS histology (cyan) without elastic image registration. B) Overlay of microCT (red) and MTS histology (cyan) after elastic image registration. Both images were aligned so that the “hole” marked with an asterisk (bottom left corner) overlap. B: Shows an almost perfect overlap of the architecture of the microCT with the structures of the MTS stained image.

**
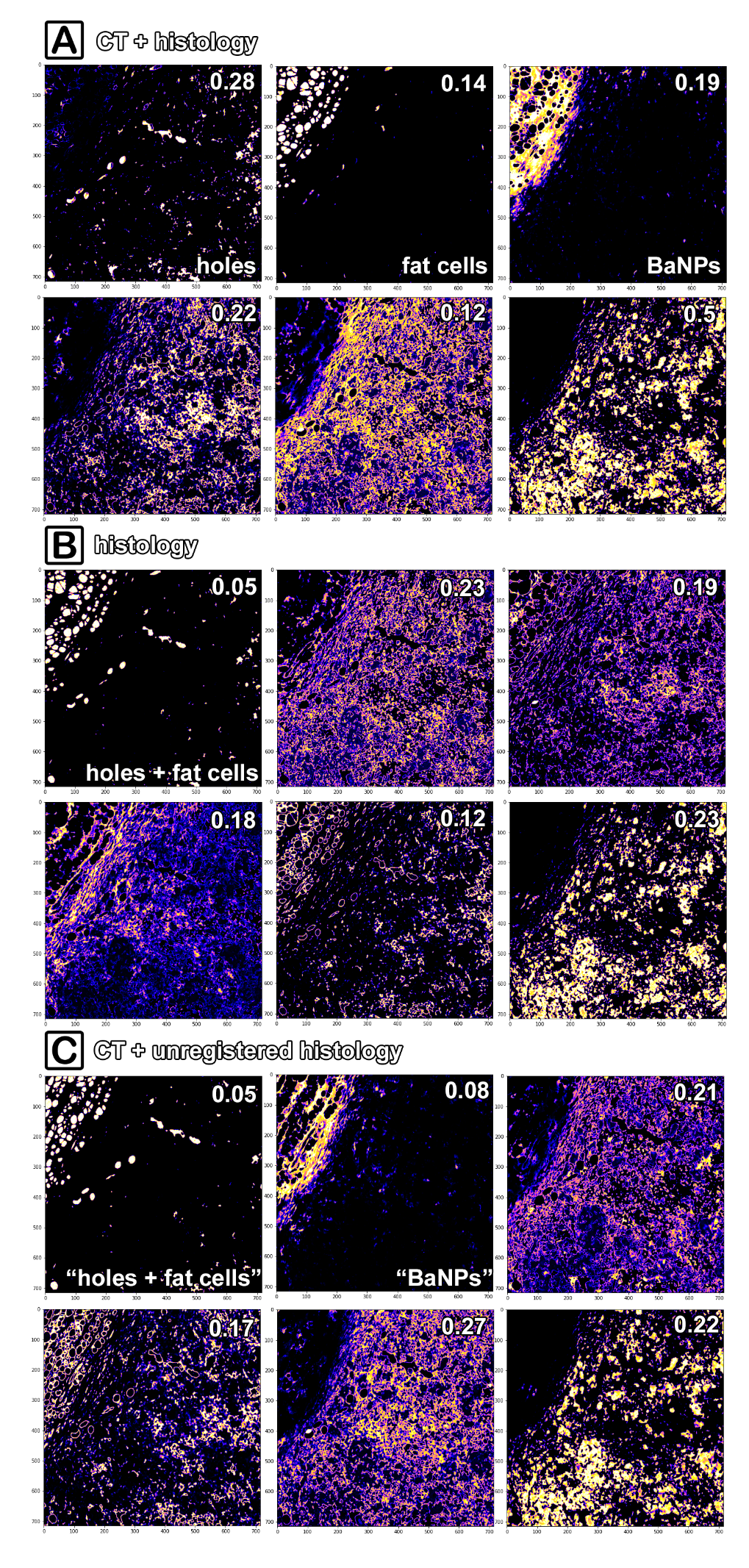
**

*Figure S2:* ***Comparison of tissue classification***

Here we show the probability maps of automatic gaussian mixed-model estimations with six classes in a four-dimensional feature space composed of the intensity in the CT data and the three colour components of the MTS histology data for each pixel position. The mixing values of each class are stated in the top right corner of each class image.

A: Shows the tissue classification as shown in Figure 6 (CT + registered histology). B: Shows the tissue classification performed just on the MTS histology. C: Shows the tissue classification performed with both modalities but without performing the elastic image registration on the histology image (CT+ unregistered histology).

The segmentation of the BaNPs, the fat cell and the porous holes inside the tissue can only be achieved when using microCT data in combination with registered histology.
